# Supplementary material for: Black carbon radiative effects highly sensitive to emitted particle size when resolving mixing-state diversity
Source: Nat Commun. 2018 Aug 27;9:3446. doi: 10.1038/s41467-018-05635-1 (PMC6110859; doi:10.1038/s41467-018-05635-1)
Supplement: Supplementary file 1 — Supplementary Information [file 41467_2018_5635_MOESM1_ESM.pdf]

Supplementary Information

Black carbon radiative effects highly sensitive to emitted particle size when resolving mixing-state diversity

Matsui et al.

Supplementary Figures

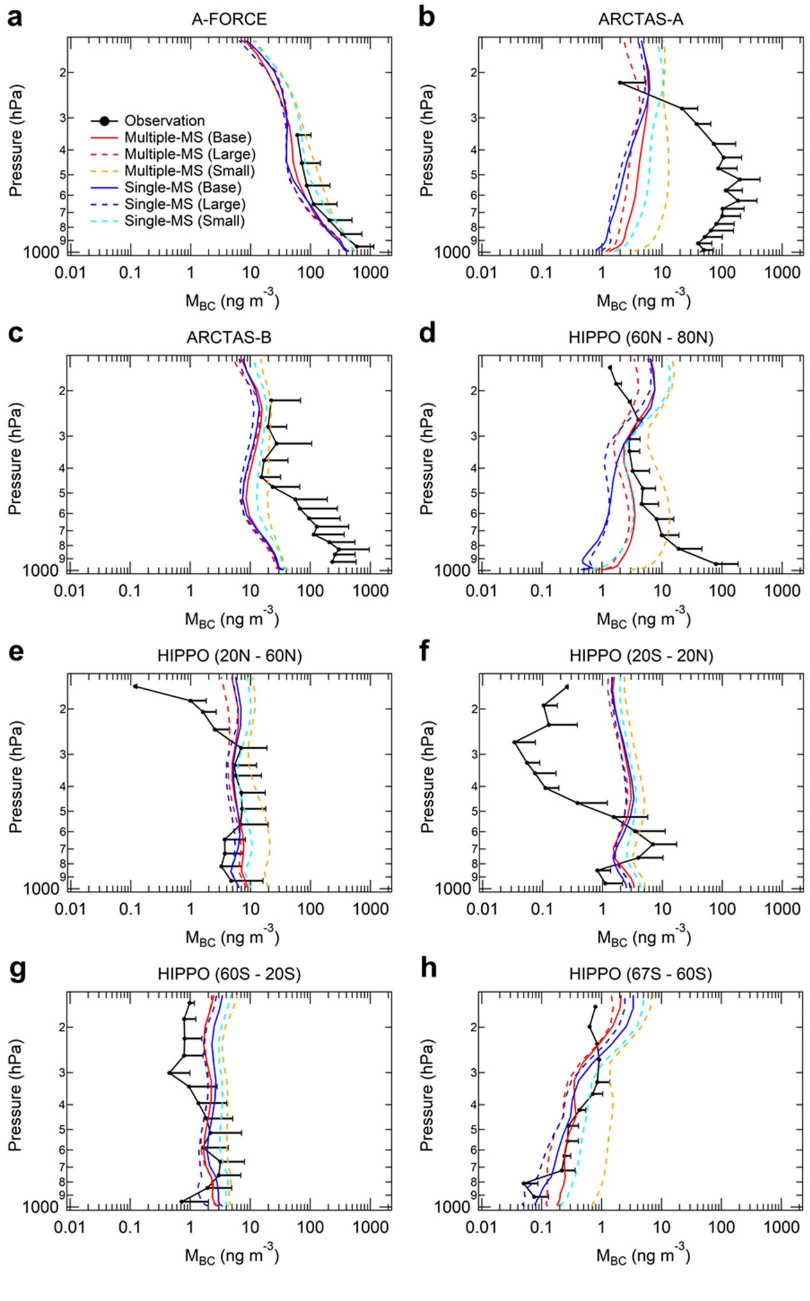

Supplementary Figure 1. Vertical profiles of black carbon mass concentrations. a-h, Vertical profiles of observed (black) and simulated (red, orange, blue, and light blue) black carbon (BC) mass concentrations during the (a) A-FORCE, (b) ARCTAS-A, (c) ARCTAS-B, and (d-h) HIPPO1 campaigns. Simulated BC mass concentrations are shown for the Base-, Large, and Small-size simulations for the Multiple-mixing-state and Single-mixing-state representations.

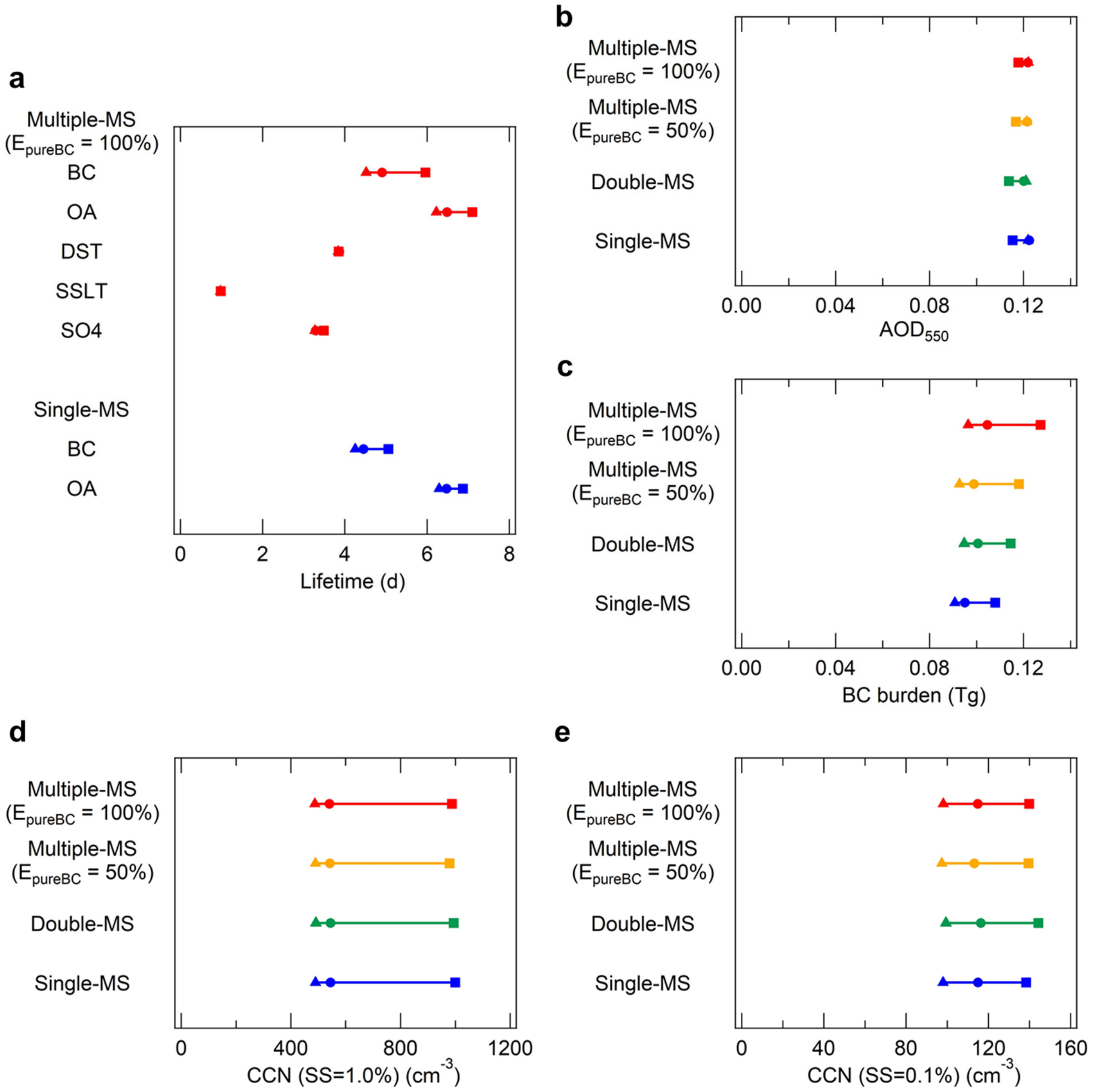

Supplementary Figure 2. Sensitivity of aerosol variables to emission size distributions. a, Lifetimes of black carbon (BC), organic aerosol, dust, sea salt, and sulfate for the Multiple-MS representation and BC and organic aerosol for the Single-MS representation. **b-e,** (b) Aerosol optical depth at 550 nm (AOD_{550}), (c) BC burden, and cloud condensation nuclei (CCN) concentrations at the surface at supersaturations of (d) 1.0% and (e) 0.1% for the four BC mixing state representations. Horizontal lines show the uncertainty ranges (**a-e**). Squares, circles, and triangles show the values in the Small, Base, and Large emission size simulations, respectively (**a-e**).

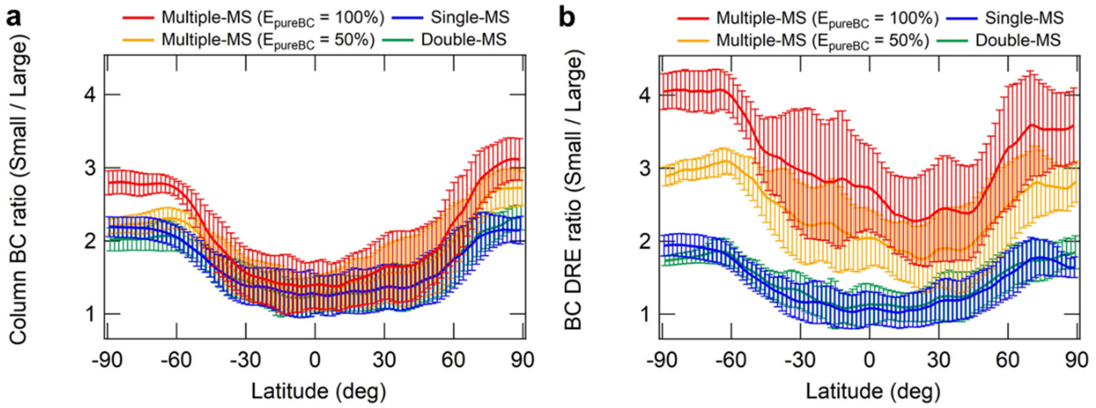

Supplementary Figure 3. Zonal-mean distributions of the sensitivity of black carbon properties. a,b, The ratio of (a) column black carbon (BC) mass concentrations and (b) BC direct radiative effect (DRE) between the Small and Large simulations for the four BC mixing state representations. Error bars show the standard deviation of longitudinal and inter-annual variability.

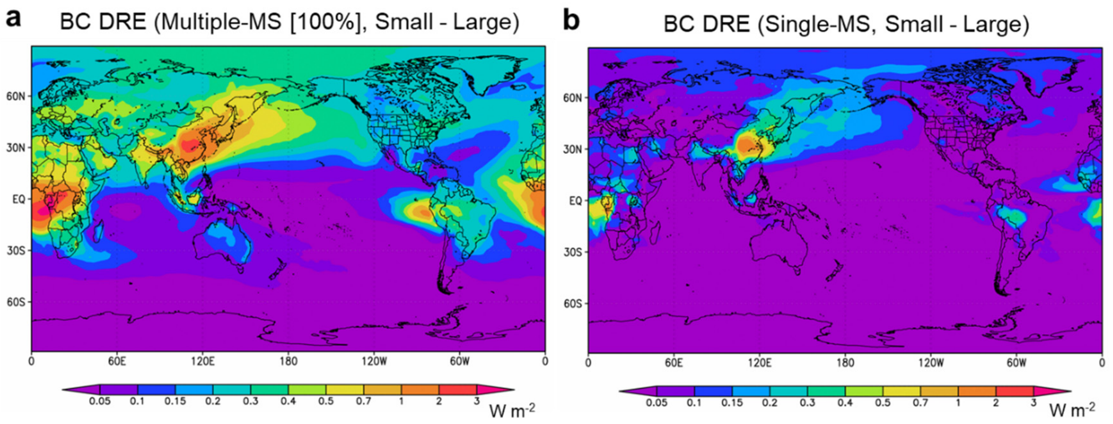

Supplementary Figure 4. Global distributions of black carbon direct radiative effect differences. a-b, The difference of black carbon (BC) direct radiative effect (DRE) between the Small and Large simulations for the (a) Multiple-mixing-state and (b) Single-mixing-state representations.

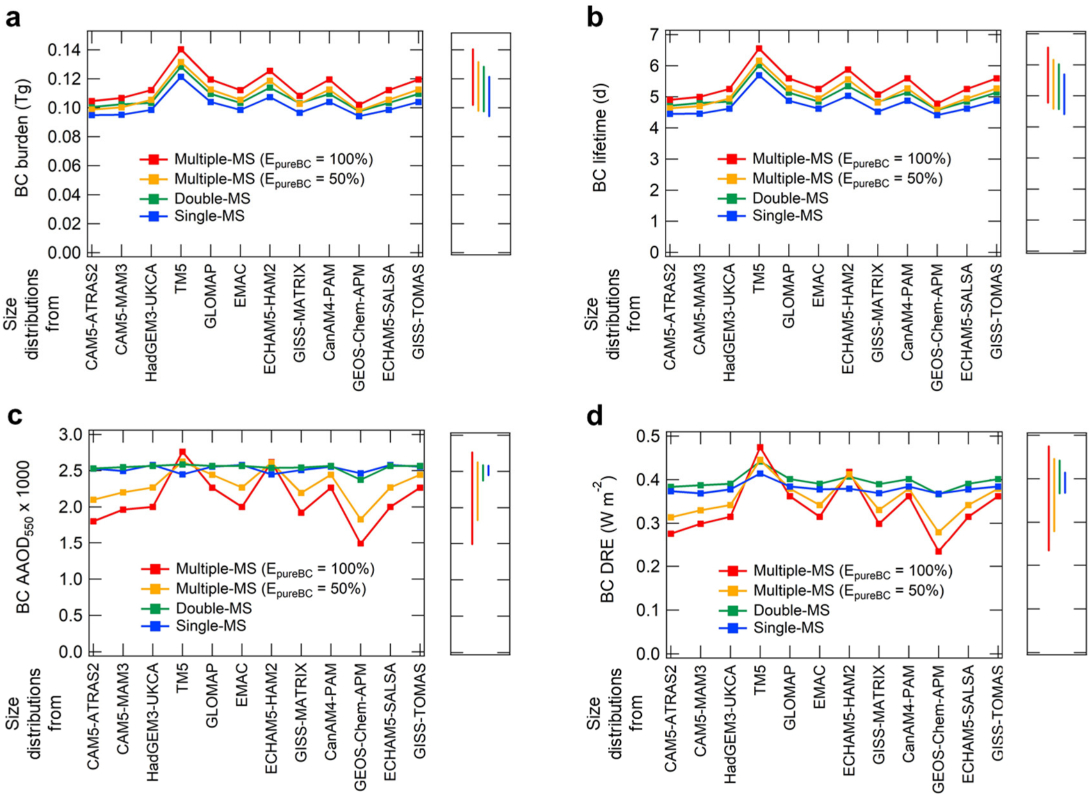

Supplementary Figure 5. Black carbon simulation results using the emission size range of the AeroCom models. a-d, Black carbon (BC) results for (a) burden, (b) lifetime, (c) absorption aerosol optical depth at 550 nm ($AAOD_{550}$), and (d) direct radiative effect (DRE) for the four BC mixing state representations. All simulations were conducted by the CAM5-ATRAS2 model, but the parameters for emission size distributions were based on the AeroCom models shown at the horizontal axis (Supplementary Table 2). Vertical bars in the small panels (for **a-d**) show the maximum-minimum range of each variable for the four BC mixing state representations.

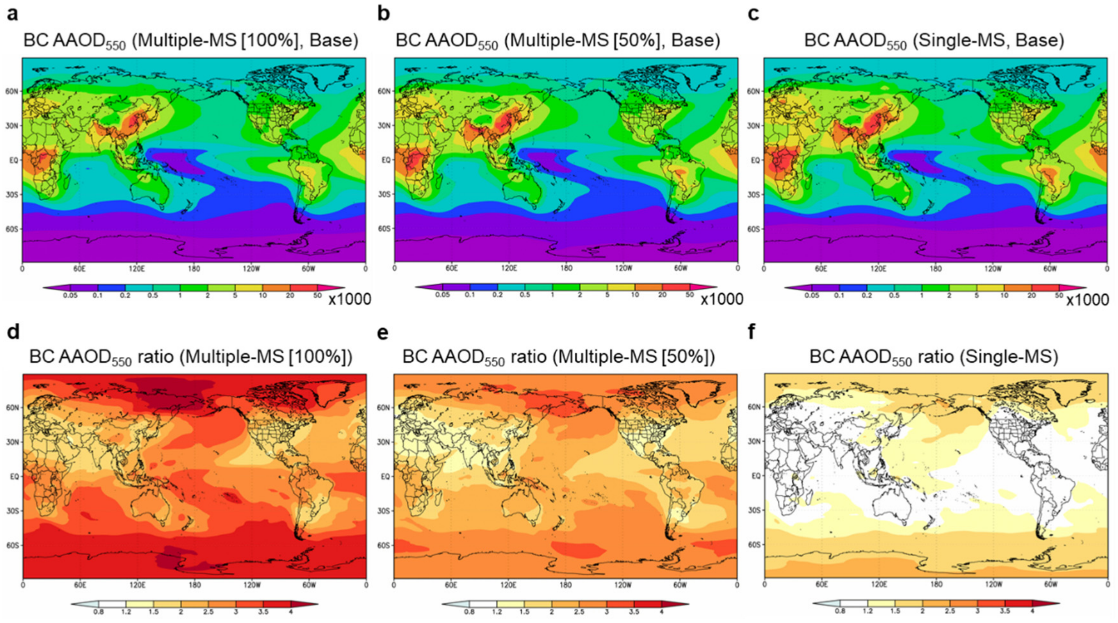

Supplementary Figure 6. Global distributions of black carbon absorption aerosol optical depth and its sensitivity. a-c, Black carbon (BC) absorption aerosol optical depth at 550 nm ($AAOD_{550}$) for the three BC mixing state representations. **d-f,** The ratio of BC $AAOD_{550}$ between the Small and Large simulations for the three BC mixing state representations.

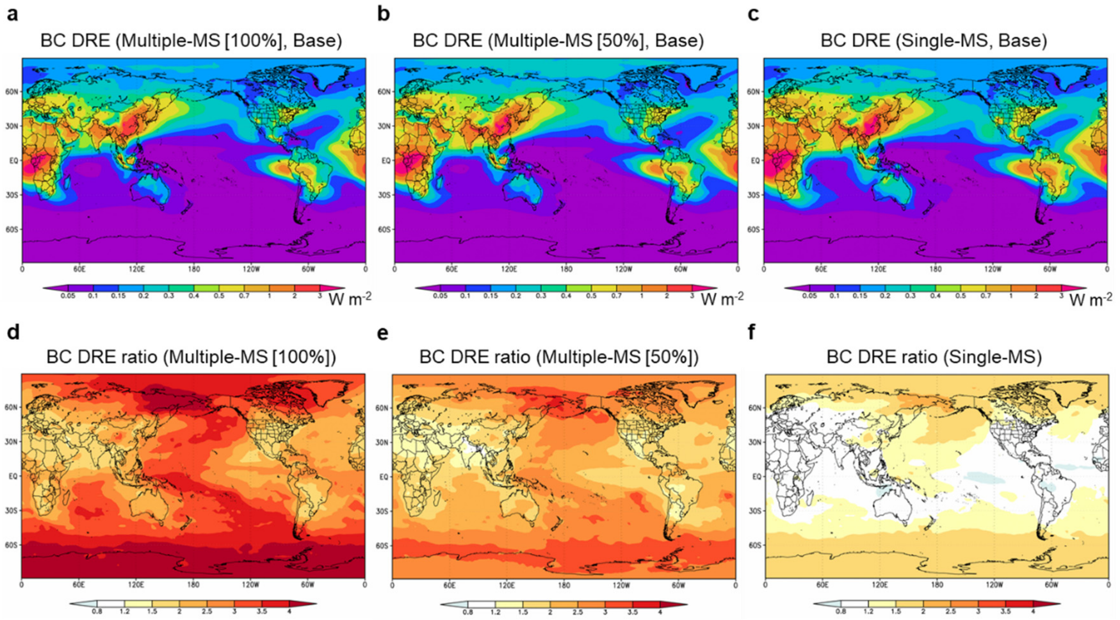

Supplementary Figure 7. Global distributions of black carbon direct radiative effect and its sensitivity. **a-c,** Black carbon (BC) direct radiative effect (DRE) for the three BC mixing state representations. **d-f,** The ratio of BC DRE between the Small and Large simulations for the three BC mixing state representations.

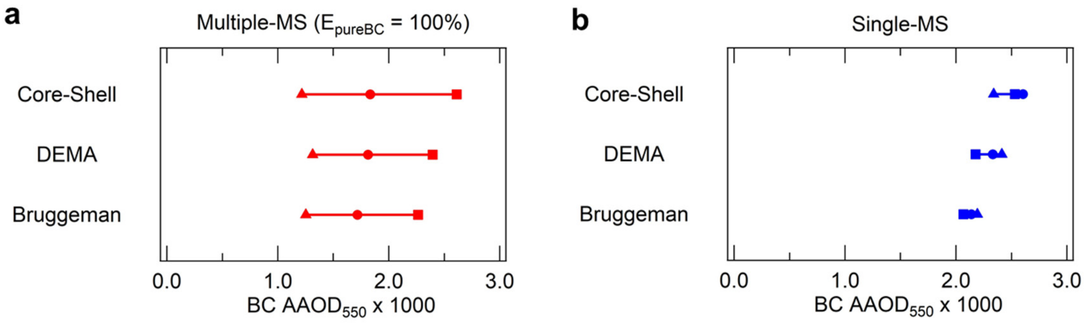

Supplementary Figure 8. Black carbon absorption aerosol optical depth for the three mixing state assumptions. a,b, The results of offline optical calculations for the Core-Shell treatment (mainly used in this study), the dynamic effective medium approximation (DEMA), and the Bruggeman mixing rule in the (a) Multple-mixing-state and (b) Single-mixing-state representations. Horizontal lines show the ranges between the Small-size and Large-size simulations. Squares, circles, and triangles show the values in the Small, Base, and Large emission size simulations, respectively.

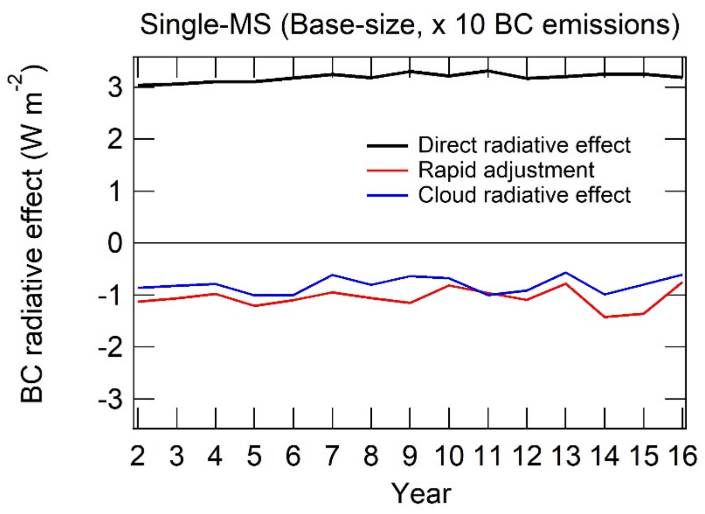

Supplementary Figure 9. Black carbon radiative effect. Direct radiative effect (black), rapid adjustment (red), and cloud radiative effect (blue) of black carbon (BC) are estimated from the simulations with 10 times enhanced BC emissions with the Single-mixing-state representation. Direct radiative effect and cloud radiative effect are instantaneous forcing of BC estimated from the definition of Ghan[1]. Rapid adjustment associated with BC is estimated from the difference between two simulations with 10 times enhanced BC emissions and without BC emissions based on Stjern et al[2] (see Methods).

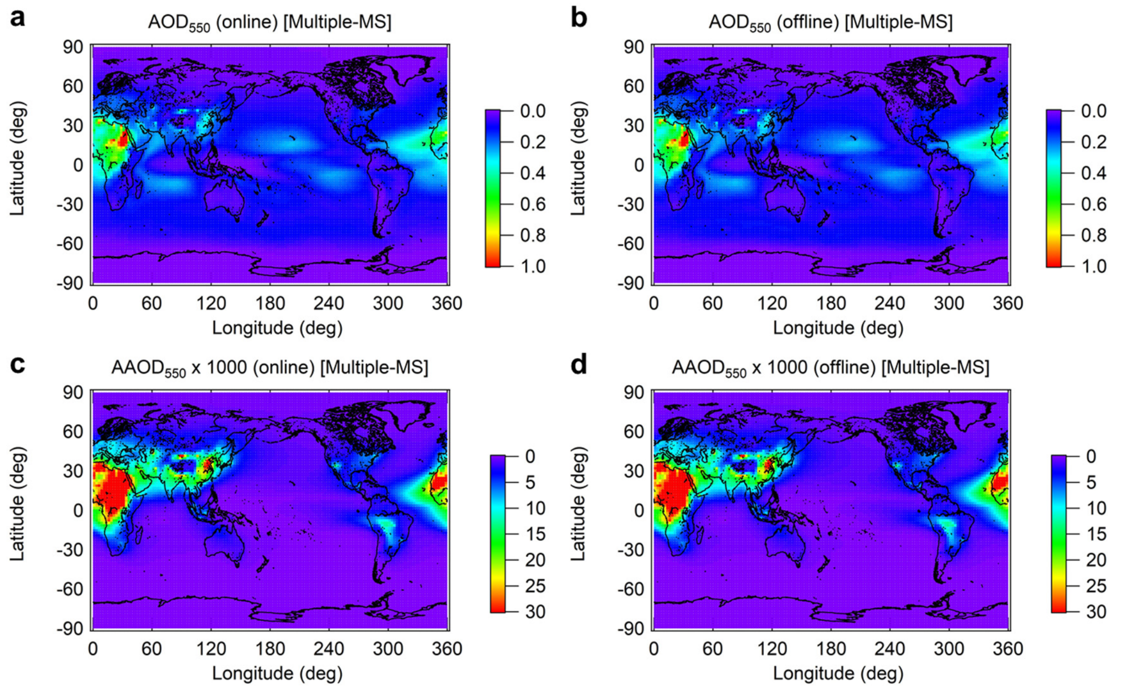

Supplementary Figure 10. Comparisons between online simulations and offline optical calculations. a-d, Global distributions of (a,b) aerosol optical depth (AOD_{550}) and (c,d) absorption aerosol optical depth ($AAOD_{550}$) for (a,c) online simulations and (b,d) offline optical calculations (Multiple-mixing-state). $AAOD_{550}$ includes contributions from both black carbon (BC) and non-BC (dust and organic aerosol). Details on optical calculations (both online simulations and offline calculations) are described in Methods.

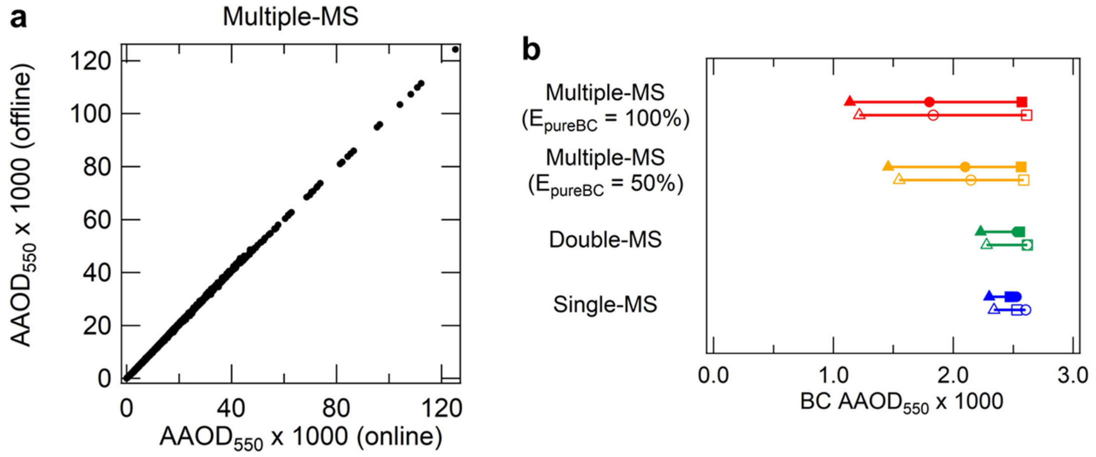

Supplementary Figure 11. Comparisons between online simulations and offline optical calculations. **a,** Scatterplot of absorption aerosol optical depth at 550 nm ($AAOD_{550}$) between online simulations and offline calculations (Multiple-mixing-state). $AAOD_{550}$ includes contributions from black carbon (BC), dust, and organic aerosol. Each point corresponds to each horizontal grid. **b**, Sensitivity of BC $AAOD_{550}$ to emission size distributions for online simulations (closed symbols) and offline calculations (open symbols). Horizontal lines show the ranges between the Small-size and Large-size simulations. Squares, circles, and triangles show the values in the Small, Base, and Large emission size simulations, respectively.

Supplementary Tables

Supplementary Table 1. Aerosol representations used in this study [a]

Model	Explanation
Multiple-MS (E_{pureBC} = 100%)	a. ***12 size*** (1-10000 nm) ***and 8 mixing state bins*** (pure BC, non-BC, and 6 different internally-mixed BC particles)<br>b. BC emissions as ***pure BC particles*** and the other emissions as ***BC-free particles***<br>c.
Multiple-MS (E_{pureBC} = 50%)	a. ***12 size*** (1-10000 nm) ***and 8 mixing state bins*** (pure BC, non-BC, and 6 different internally-mixed BC particles)<br>b. Emissions as ***pure BC (50% of total BC mass), internally-mixed BC (50% of total BC mass), and non-BC particles***<br>c. Shell (total) to core (BC) diameter ratio of 1.1 (FF sources) and 1.4 (BF/BB sources) for emissions of internally-mixed BC particles
Double-MS	a. ***12 size*** (1-10000 nm) ***and 2 mixing state bins*** (thinly-coated BC (BC mass fraction > 0.9) and others)<br>b. BC emissions as ***pure BC particles*** and the other emissions as ***BC-free particles***
Single-MS	a. ***12 size*** (1-10000 nm) and ***1 mixing state bins*** (internally-mixed BC particles only)<br>b. Emissions as ***internally-mixed BC particles***

[a] Multiple-MS (E_{pureBC} = 100%) and Single-MS are mainly used in this study.

Supplementary Table 2. Parameters of aerosol number size distributions in emissions used in this study [a]

Simulation	Median diameter		Sigma	Comment
	FF	BF/BB		
Base	70	100	1.8	Matsui[3] and Matsui and Mahowald[4]
Large	80	200	1.8	Lee et al.[5] and Carslaw et al.[6]
Small	30	50	1.8	Lee et al.[5] and Carslaw et al.[6]
Sens 1	80	80	1.8	CAM5-MAM3 [7]
Sens 2	60	150	1.59	HadGEM3-UKCA [8], EMAC [9], and ECHAM5-SALSA [10]
Sens 3	30	30	1.59	TM5 [11]
Sens 4	30	80	1.8	GLOMAP [12,13], CanAM4-PAM [14], and GISS-TOMAS [15]
Sens 5	60	60	1.59	ECHAM5-HAM2 [16]
Sens 6	50	100	1.8	GISS-MATRIX [17]
Sens 7	60	150	1.8	GEOS-Chem-APM [18]

[a] Aerosol emissions are assumed to have lognormal size distributions.

Supplementary Table 3. Summary of global-mean BC statistics obtained in this study

Parameter	Unit	Multiple-MS (E_{pureBC} = 100%)			Single-MS		
		Base	Large	Small	Base	Large	Small
Online simulation							
Emission	Tg y^{-1}	7.8	7.8	7.8	7.8	7.8	7.8
Burden	Tg	0.10	0.096	0.13	0.095	0.091	0.11
Lifetime	d	4.9	4.5	6.0	4.5	4.3	5.1
$AAOD_{550}$	×1000	1.8	1.1	2.6	2.5	2.3	2.5
MAC	m^2 g^{-1}	8.8	6.0	10.3	13.6	12.9	11.7
DRE	W m^{-2}	0.28	0.18	0.42	0.37	0.35	0.38
Offline optical calculation							
$AAOD_{550}$ (core+shell)	×1000	1.8	1.2	2.6	2.6	2.3	2.5
$AAOD_{550}$ (core only)	×1000	1.2	0.79	1.5	1.1	1.0	1.1
$E_{abs,coat}$	---	1.6	1.5	1.8	2.4	2.3	2.2
MAC_{core}	m^2 g^{-1}	5.6	4.2	5.9	5.9	5.7	5.4
$AAOD_{550}$ (DEMA [a])	×1000	1.8	1.3	2.4	2.3	2.4	2.2
$AAOD_{550}$ (Bruggeman [b])	×1000	1.7	1.3	2.3	2.1	2.2	2.1

[a] The dynamic effective medium approximation[19-21] (see Methods).
[b] The Bruggeman mixing rule[20] (see Methods).

Supplementary Table 4. Direct radiative effect, rapid adjustment, and cloud radiative effect of BC estimated from simulations with 10 times enhanced BC emissions

Parameter	Unit	Base	Large	Small
Direct radiative effect	W m^{-2}	3.2 ± 0.082	2.7 ± 0.048	3.9 ± 0.087
Rapid adjustment	W m^{-2}	-1.1 ± 0.19	-0.71 ± 0.25	-1.8 ± 0.32
Cloud radiative effect	W m^{-2}	-0.81 ± 0.16	-0.48 ± 0.15	-1.2 ± 0.14
Rapid adjustment / DRE	---	-0.32 ± 0.060	-0.25 ± 0.095	-0.44 ± 0.080

Averages and standard deviations of 15-year simulations (Single-mixing-state) are shown (see Methods).

Supplementary References

1. Ghan, S. J. Technical note: Estimating aerosol effects on cloud radiative forcing. *Atmos. Chem. Phys.* **13**, 9971-9974 (2013).
2. Stjern, C. W. *et al.* Rapid adjustments cause weak surface temperature response to increased black carbon concentrations. *J. Geophys. Res. Atmos.* **122**, 11462-11481 (2017).
3. Matsui, H. Development of a global aerosol model using a two-dimensional sectional method: 1. Model design. *J. Adv. Model. Earth Syst*. **9**, 1921-1947 (2017).
4. Matsui, H. & Mahowald, N. Development of a global aerosol model using a two-dimensional sectional method: 2. Evaluation and sensitivity simulations. *J. Adv. Model. Earth Syst*. **9**, 1887-1920 (2017).
5. Lee, L. A. *et al.* The magnitude and causes of uncertainty in global model simulations of cloud condensation nuclei. *Atmos. Chem. Phys.* **13**, 8879-8914 (2013).
6. Carslaw, K. S. *et al*. Large contribution of natural aerosols to uncertainty in indirect forcing. *Nature* **503**, 67-71 (2013).
7. Liu, X. *et al.* Toward a minimal representation of aerosols in climate models: description and evaluation in the Community Atmospheric Model CAM5. *Geosci. Model Dev.* **5**, 709-739 (2012).
8. Mann, G. W. *et al.* Intercomparison and evaluation of global aerosol microphysical properties among AeroCom models of a range of complexity. *Atmos. Chem. Phys.* **15**, 4679-4713 (2014).
9. Pringle, K. J. *et al.* Description and evaluation of GMXe: a new aerosol submodel for global simulations (v1). *Geosci. Model Dev.* **3**, 391-412 (2010).
10. Bergman, T. *et al.* Evaluation of the sectional aerosol microphysics module SALSA implementation in ECHAM5-HAM aerosol-climate model. *Geosci. Model Dev*. **5**, 845-868 (2012).
11. Aan de Brugh, J. M. J. *et al.* The European aerosol budget in 2006. *Atmos. Chem. Phys.* **11**, 1117-1139 (2011).
12. Spracklen, D. V. *et al.* A global off-line model of size-resolved aerosol microphysics: I. Model development and prediction of aerosol properties. *Atmos. Chem. Phys.* **5**, 2227-2252 (2005).
13. Mann G. W. *et al.* Intercomparison of modal and sectional aerosol microphysics representations within the same 3-D global chemical transport model. *Atmos. Chem. Phys.* **12**, 4449-4476 (2012).
14. von Salzen, K. Piecewise log-normal approximation of size distributions for aerosol modeling. *Atmos. Chem. Phys.* **6**, 1351-1372 (2006).
15. Lee, Y. H. & Adams, P. J. Evaluation of aerosol distributions in the GISS-TOMAS global aerosol microphysics model with remote sensing observations. *Atmos. Chem. Phys*. **10**, 2129-2144 (2010).
16. Zhang, K. *et al.* The global aerosol-climate model ECHAM-HAM, version 2: sensitivity to improvements in process representations. *Atmos. Chem. Phys.* **12**, 8911-8949 (2012).
17. Bauer, S. E. *et al.* MATRIX (Multiconfiguration Aerosol TRacker of mIXing state): An aerosol microphysical module for global atmospheric models. *Atmos. Chem. Phys.* **8**, 6003-6035 (2008).
18. Yu, F. & Luo, G. Simulation of particle size distribution with a global aerosol model: contribution of nucleation to aerosol and CCN number concentrations. *Atmos. Chem. Phys.* **9**, 7691-7710 (2009).
19. Chylek, P., Ramaswamy, V. & Cheng, R. J. Effect of graphitic carbon on the albedo of clouds. *J. Atmos. Sci.* **41**, 3076-3084 (1984).
20. Jacobson, M. Z. Effects of externally-through-internally-mixed soot inclusions within clouds and precipitation on global climate. *J. Phys. Chem. A* **110**, 6860-6873 (2006).
21. Fierce, L., Bond, T. C., Bauer, S. E., Mena, F. & Riemer N. Black carbon absorption at the global scale is affected by particle-scale diversity in composition. *Nat. Commun.* **7**, 12361 (2016).
